# Thermal and mechanical reinforcement of a novel paraffin-based hydroxyl-terminated polybutadiene (HTPB) binder containing a three-dimension (3D) diurea–paraffin wax (DU–PW) for prevention of PW leakage†

**DOI:** 10.1039/c7ra10574f

**Published:** 2018-01-03

**Authors:** Xia Gao, Tianbo Zhao, Guan Luo, Baohui Zheng, Hui Huang, Xue Han, Rui Ma, Yuqiao Chai

**Affiliations:** Key Laboratory of Cluster Science of Ministry of Education, Beijing Institute of Technology Beijing 100081 PR China bit_bipt@126.com +86-10-81381389; Institute of Chemical Materials, China Academy of Engineering Physics (CAEP) Mianyang 621900 PR China

## Abstract

Leakage of paraffin wax (PW) is a major concern in the development of polymer bonded explosive (PBX) systems because it relates to the amount of PW that can be used as a desensitizer or a fuel, which, in turn, affects the mechanical performance and tolerance of PBX in high-temperature environments. Hydroxyl-terminated polybutadiene (HTPB) binders significantly contribute desirable polymer features to PBX. Thus, a three-dimension (3D) high-temperature non-flowing diurea–paraffin wax (DU–PW) composite was synthesized and creatively employed to a HTPB binder. DU–PW/PW/HTPB composites with different contents of the 3D DU–PW phase change material (PCM) were prepared through a cast molding process. The differential scanning calorimetry (DSC) results demonstrate that these composites can show high phase-change enthalpies and good thermal reliability. As observed from the scanning electron microscope (SEM) photographs, adding DU–PW can clearly reduce the number of holes caused by the leaked PW on the fracture surface of DU–PW/HTPB. Moreover, the addition of DU–PW can remarkably reduce the leakage of PW and improve the thermal stability as well as mechanical properties of the PW-based HTPB. These observations present the potential of utilizing form-stable PCM (FSPCM) to solve the problem of PW leakage in PBX systems.

## Abstract 2

DU is creatively used as supporting materials to shape-stablize PW. The prepared DU–PW composite can effectively improve the thermal and mechanical properties of PW/HTPB, indicating a prospect to prevent PW leakage in PBX.

## Graphical abstract

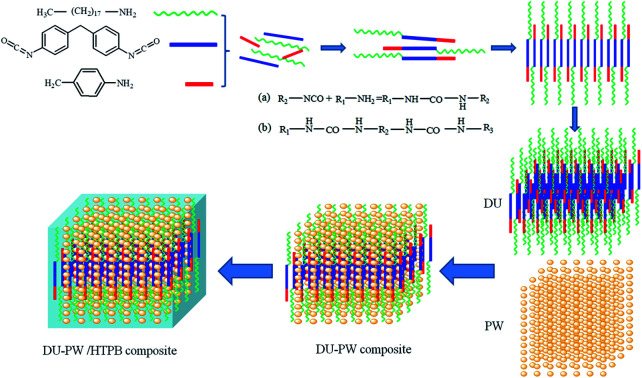

## 1. Introduction

Polymer bonded explosives (PBX) are promoted for insensitive munitions, consisting of explosive crystals, polymer binders, desensitizers, plasticizers, thermal stabilizers and some other functional additives.^1–4^ Polymer binders play an important role as structural supporting materials and endow PBX with desirable properties of polymers, such as cast-ability and machine-ability.^5,6^ In the past few decades, hydroxyl-terminated polybutadiene (HTPB) has been widely used as a binder in military engineering due to its superior mechanical and ballistic properties.^7,8^ In addition, HTPB-derived polyurethane can provide a matrix of networks to impart dimensional stability and structural integrity to PBX.^9^ With the increasing attention on the trade-off between high performance and low sensitivity of energetic materials,^10,11^ paraffin wax (PW) serves as a promising desensitizer and solid fuel^12^ due to its low cost, controllable phase-transition temperature, chemical stability, environment-friendliness, high heat of combustion and burning regression rate.^12–15^ Jin *et al.*^16^ studied the coating and desensitization effects of PW/stearic acid (SA) composite on 2,4,6,8,10,12-hexanitro-2,4,6,8,10,12-hexaazaisowurtzitane (HNIW). Kawachi *et al.*^13^ investigated the thermal and mechanical tests of a combustible grain (PW/HTPB) casted with maximum loading; the results indicated that this material could meet the criteria of performance and safety required for this type of a combustible grain. However, poor mechanical properties, diffusion and leakage of melted PW will always be an issue if the PW is directly suspended in HTPB using traditional methods.^17^ Therefore, prevention of PW leakage is the main topic of most of these investigations.

PW is usually categorized as an organic solid–liquid phase change material (PCM). In recent years, microencapsulated PCM (MEPCM) and form-stable PCM (FSPCM) have proved to be effective techniques to prevent the leakage in PCM. Compared with FSPCM, MEPCM with shell materials requires complicated synthesis procedures, high volume change, large super-cooling and high cost, which hinder its application.^18–20^ FSPCM is prepared by absorbing PCM with various polymers and inorganic porous materials as supporting materials, including polyethylene glycol (PEG),^21^ high density polyethylene (HDPE),^22^ epoxy,^23^ polyurethane (PU) and PU foam,^24–26^ porous metal fiber sintered felt (PMFSF),^27^ multi-walled carbon nanotubes (MWCNTs),^28^ graphene,^29^ nanoplatelet graphite,^30^ activated carbon (AC),^31^ expanded graphite (EG)^32^ and fibers.^33,34^ Samui *et al.*^35^ developed a microwave technology to prepare a shape-stabilized PEG-cellulose acetate blend with high PEG loading. Karaipekli *et al.*^36^ prepared an expanded perlite (EP)/paraffin composite PCM with highly enhanced thermal conductivity using carbon nanotubes. Although these supporting materials can effectively shape-stabilize PW during its phase transition, the temperature range required to keep PW form-stable is too narrow for its application in higher-temperature environments. Lubricating grease, a type of high-temperature material such as cream, is generally prepared by thickening base oil with polyurea.^37^ Thus, our team introduced diurea (DU) as a new supporting material to absorb PW owing to its simple preparation process, high stability, amphipathy and surface activity.

In order to find a solution to PW leakage in HTPB binders and inspired by the recent progress in FSPCM, a high-temperature DU–PW composite is creatively employed to the HTPB matrix. In this study, it is meaningful to investigate the effect of different loadings of the DU–PW composite on the leakage, thermal and mechanical properties of the DU–PW/PW/HTPB composite because these HTPB binder composites with stable shapes can be employed for the practical application of PBX systems.

## 2. Experiment

### 2.1. Materials

Paraffin wax (*T*_m_ = 50–52 °C) was purchased from Shanghai Huashen Rehabilitation Material Co., Ltd., China. Octadecylamine (CP) was supplied by the Research Institute of Tianjin Guangfu Fine Chemical Industry Co., Ltd., China. Paratoluidine (AR) was commercially obtained from Shanghai Macklin Chemical Co., Ltd., China. Bis(4-isocyanatophenyl)methane (MDI, 98%) was supplied by J&K Chemical Co., Ltd., China. Petroleum ether (AR) was purchased from Chongqing Chuandong Chemical Co., Ltd., China.

Hydroxylterminated polybutadiene (HTPB) (OH value = 0.76 mmol g^−1^) was purchased from Liming Research & Design Institute of Chemical Industry Co., Ltd., China. Toluene diisocyanate (TDI) (AR) was supplied by Xiya Chemical Industry Co., Ltd., China. Dinoctylsebacate (DOS) (AR) was purchased from Jiangyin Bolong Chemical Group Co., Ltd.

### 2.2. Preparation of DU–PW composite

The preparation process of the DU–PW composite can be described as follows. Octadecylamine and paratoluidine were mixed with partial PW at 79 °C. Simultaneously, MDI was exhaustively blended with PW in a container at 79 °C. Then, the MDI blend was poured into an organic amine mixture quickly and agitated at 99 °C for 0.5 h. Subsequently, the temperature of the oil bath increased to 190 °C at the rate of 2 °C min^−1^. The mixture was continuously agitated for 1 h and naturally cooled to room temperature. Finally, the DU–PW composite (DU/PW = 2/8) was prepared. The photographs of pristine PW and the prepared DU–PW composite are shown in Fig. S1.† To obtain the DU particles without PW, DU–PW was extracted with petroleum ether for 48 h, repeatedly centrifuged, and dried at 25 °C for 24 h.

### 2.3. Preparation of DU–PW/PW/HTPB composites

In the preparation process of the DU–PW/PW/HTPB composite, the DU–PW composite was intended to replace 20 wt%, 40 wt%, 60 wt%, and 100 wt% of PW in the HTPB matrix. The HTPB (324.17 g) was blended with DOS (154.95 g), TDI (20.88 g), PW and DU–PW at 60 °C for 30 min. The defined amount of PW and DU–PW is presented in Table S1.† Then, the mixture was placed in a vacuum oven at 60 °C for 30 min to remove air bubbles. Subsequently, the mixture was poured into aluminum molds with different specimen dimensions. Finally, these molds were cured in an oven at 65 °C for 3 days and cooled to room temperature. The photographs of pristine HTPB, the PW/HTPB composite and the DU–PW/HTPB composite are shown in Fig. S2.†

### 2.4. Characterizations

The chemical composition of the DU–PW and DU–PW/PW/HTPB composites was investigated by Fourier transformed infrared spectroscopy (FT-IR, Nicolet 6700), within a wave number range of 350–4000 cm^−1^.

The microstructure of the DU particles and the DU–PW/PW/HTPB composite samples was studied using a scanning electron microscope (SEM, ZEISS ULTRA 55). The DU–PW/PW/HTPB samples were prepared by cryofracturing in liquid nitrogen. The cross sections of the composites and the DU particles were sputter-coated with gold before analysis.

The thermal stability of the DU–PW and DU–PW/PW/HTPB composites was studied using a thermal gravimetric analyzer (TGA, TG-DTA 6200 LAB SYS) with a heating rate of 10 °C min^−1^ from 25 °C to 600 °C in a stream of nitrogen.

The phase change properties of DU–PW and DU–PW/PW/HTPB composites were studied using a differential scanning calorimeter (DSC, DSC Q2000 V24.11 Build 124) with a temperature range from −10 to 75 °C at a heating rate of 10 °C min^−1^, in a nitrogen stream. An accelerated 500 thermal cycling test (25–75 °C) was conducted in a programmable high-low temperature test chamber.

The compression test of the DU–PW/PW/HTPB samples with dimensions of *Φ* 20 mm × 20 mm was characterized according to ISO 7743:2007 using an MTS Criterion Model 45 electromechanical universal testing machine. The crosshead speed was set to 5 mm min^−1^. For each sample, the average value of five data was taken.

The tensile test of the DU–PW/PW/HTPB samples was measured on a MTS Criterion Model 45 electromechanical universal testing machine with a crosshead speed of 5 mm min^−1^ according to ISO 37:2005.

Leaking test on the samples was performed in a cylinder placed in an oven for 30 days at 75 °C. The samples with dimension of *Φ* 24 mm × 125.75 mm were packed using a filter paper. To get a quantitative value of the PW leakage, the initial and final weights of the samples and the filter paper were recorded and calculated.

## 3. Results and discussions

### 3.1. Scheme and structure of DU–PW/HTPB composite

**Fig. 1:**
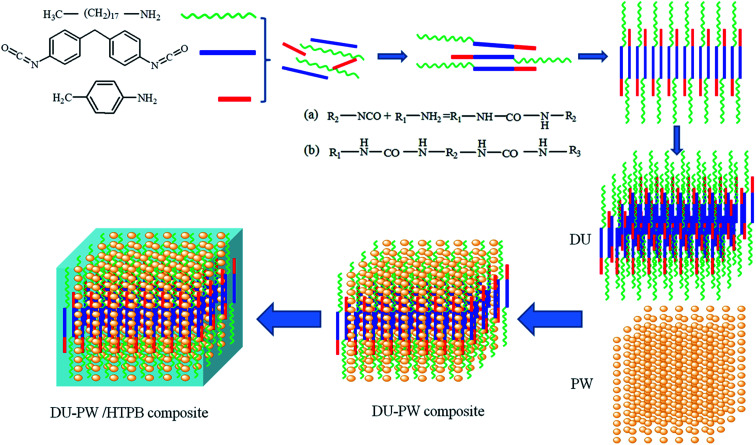
Schematic illustration of the synthesis process of DU–PW/HTPB binder composite: R_1_, R_3_ are alkyl, cycloalkyl or aryl; R_2_ is arylene, alkylene or cycloalkylene.

The schematic illustration of the synthesis process of the DU–PW/HTPB binder composite is shown in Fig. 1. As presented in Fig. 1, the organic amines (octadecylamine and paratoluidine) react with MDI and produce the DU molecules. The DU molecules self-assemble into a three-dimensional (3D) supramolecular structure by intermolecular interaction. Then, the DU–PW composite was synthesized by absorbing PW into the 3D supramolecular structure of DU. Subsequently, the DU–PW composite was blended with the HTPB matrix to prepare the DU–PW/HTPB composite. In this composite, the 3D supramolecular structure of DU is formed like a cage to lock the PW molecules, which is intended to stabilize the PW. Thus, the process of introducing DU–PW into the HTPB matrix can prevent the leakage of PW.

### 3.2. Chemical composition of DU–PW and DU–PW/HTPB composites

**Fig. 2:**
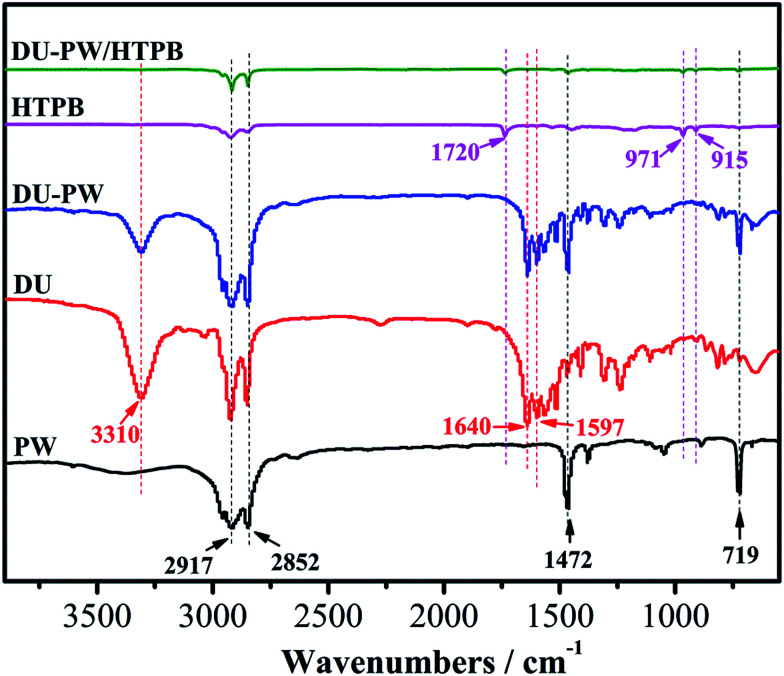
FTIR spectra of pristine PW, DU, DU–PW composite, pristine HTPB and DU–PW/HTPB composites.

The FTIR spectra of PW, DU, DU–PW, HTPB and DU–PW/HTPB composites are presented in Fig. 2. As observed in Fig. 2, in the spectrum of PW, the peaks at 2917 and 2852 cm^−1^ were attributed to the aliphatic C–H stretching vibration of the –CH_3_ and –CH_2_ groups,^38^ respectively. The peaks at 1462 and 719 cm^−1^ were ascribed to the bending and in-plane rocking vibration of the CH_2_ groups.^39^ For the DU sample, the absorption peak at 3310 cm^−1^ was assigned to the stretching vibration of the hydrogen bonded N–H group. The peak located at 1597 cm^−1^ was assigned to the bending vibration absorption of the N–H group. The characteristic absorption peak of –C=O group was found at 1640 cm^−1^, due to the hydrogen bond between the DU molecules. In the spectrum of the DU–PW composite, the characteristic peaks of PW and DU can be observed. This indicated that the DU–PW composite has been successfully synthesized without any chemical reaction. For HTPB, the peak at 1720 cm^−1^ belonged to the urethane groups and the absorption peaks at 971 and 915 cm^−1^ were corresponding to the bending vibrations of the C=C band.^6^ Compared with pristine HTPB, the spectrum of the DU–PW/HTPB composite was almost consistent, suggesting that no chemical interaction occurred between the two components. The results indicated that the DU–PW/HTPB composite had been prepared through physical blending.

### 3.3. Morphology of DU and DU–PW/PW/HTPB composites

**Fig. 3:**
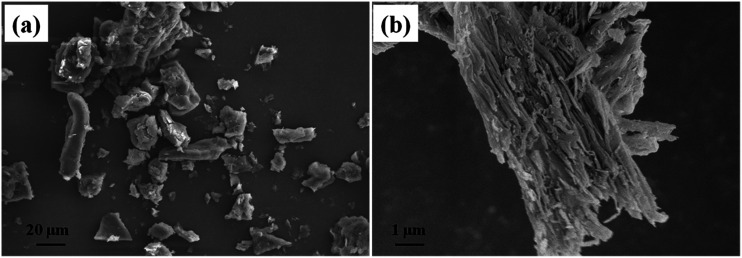
SEM micrographs of DU particles.

**Fig. 4:**
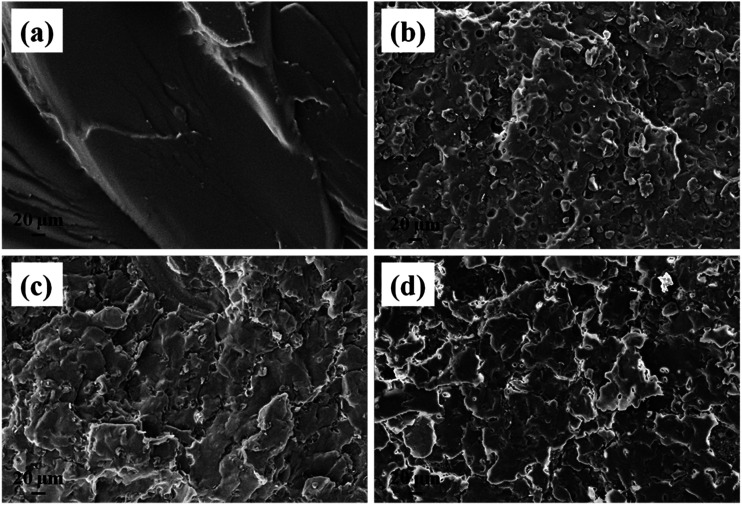
SEM micrographs of (a) pristine HTPB, (b) PW/HTPB, (c) 60 wt% DU–PW/PW/HTPB and (d) DU–PW/HTPB composites.

The morphology of the DU particles without PW is shown in Fig. 3. In Fig. 3a, the DU particles exhibited an irregular shape with a diameter range of about 10–100 μm. As observed from Fig. 3b, there are numerous 3D networks of fibrous and strip-like structures existing in DU particles, which would trap the movement of melted PW and support PW to FSPCM. Therefore, the DU particles with a gel network structure could serve as a new supporting material.^40^ The microstructures of the fracture surfaces of the HTPB and DU–PW/PW/HTPB composites are shown in Fig. 4. As shown in Fig. 4a, there are some rupture cracks on the smooth fracture surface of pristine HTPB due to the brittle failure of HTPB in liquid nitrogen. For the PW/HTPB composite, numerous dark holes and spherical particles are distributed on the fracture surface. These holes may be left by the exuded PW particles, which were displayed as spherical particles. With the addition of the DU–PW composite, fewer holes and PW particles could be observed on the fracture surface of the DU–PW/PW/HTPB composite since the self-assembled 3D DU could make a thickened entanglement with the pure PW and HTPB.^40^ Therefore, few holes and PW particles exist on the fracture surface of the DU–PW/HTPB composite. This indicated that the addition of the DU–PW composite can effectively prevent the separation of PW from the HTPB matrix.

### 3.4. Phase change properties of DU–PW/PW/HTPB composites

**Fig. 5:**
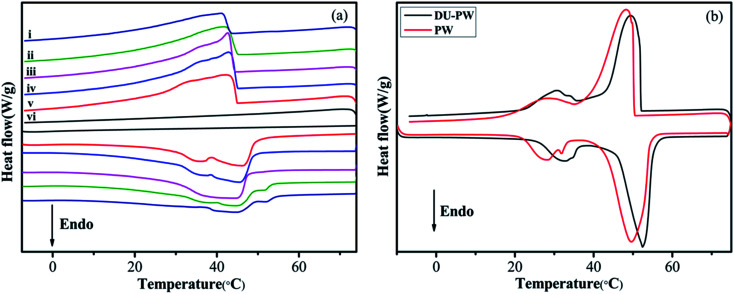
DSC curves of (a-i) DU–PW/HTPB, (a-ii) 60 wt% DU–PW/PW/HTPB, (a-iii) 40 wt% DU–PW/PW/HTPB, (a-iv) 20 wt% DU–PW/PW/HTPB, (a-v) PW/HTPB, and (a-vi) pristine HTPB, (b) PW and DU–PW composites.

**Fig. 6:**
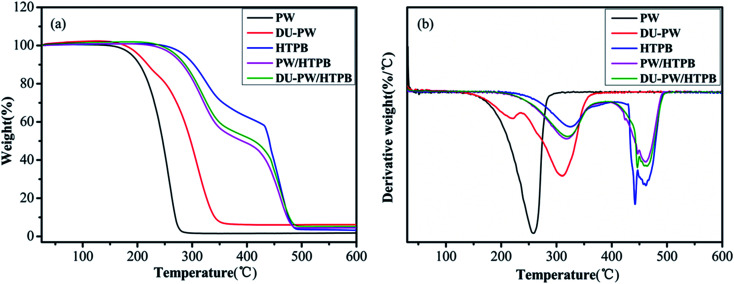
(a) TGA and (b) DTG curves of PW, DU–PW, pristine HTPB, PW/HTPB and DU–PW/HTPB composites.

The phase change properties of PW, DU–PW, pristine HTPB, PW/HTPB and DU–PW/PW/HTPB composites are presented in Fig. 6 and summarized in Table S2.† As displayed in Fig. 5a, there is no phase change peak on the DSC curve of the HTPB matrix, indicating that no phase change occurred in HTPB within this temperature range. For the DU–PW/PW/HTPB composites, phase change occurred during the melting and freezing processes, caused by the addition of PW and DU–PW. In Fig. 5b, the temperatures at the endothermic peak (*T*_m_) and exothermic peak (*T*_c_) of DU–PW were improved compared with PW due to the shape-stabilizing effect of DU on PW. This would delay the phase change process of PW. As listed in Table S2,† the difference (Δ*T*_s_) between the *T*_m_ and *T*_c_ of DU–PW/PW/HTPB composites was lower than that of PW/HTPB, indicating that DU–PW can limit the super-cooling phenomena of PW-based HTPB. As the content of DU–PW increased, the calculated phase change enthalpies (Δ*H*_2_) of DU–PW/PW/HTPB composites decreased because the total content of pure PW in HTPB decreased. In addition, the Δ*H*_1_ of most DU–PW/PW/HTPB composites was lower than Δ*H*_2_; owing to that the curing process (65 °C, 3 days) would lead to the diffusion of PW in the composites. However, the Δ*H*_1_ of 20 wt% and 40 wt% DU–PW/PW/HTPB composites was slightly higher than that of PW/HTPB. It helps to illustrate that the DU can hold the PW firmly in the HTPB matrix. Moreover, the difference (error%) between Δ*H*_1_ and Δ*H*_2_ of the DU–PW/PW/HTPB composites was much lower than that of PW/HTPB, implying that less diffusion of PW took place in DU–PW/PW/HTPB than that in PW/HTPB. This indicates that the DU–PW/PW/HTPB composites exhibit high heat capacity and higher form-stability than PW/HTPB due to the shape-stabilizing function of DU–PW.

### 3.5. Thermal stability of DU–PW/PW/HTPB composites

TGA was used to investigate the thermal stability of the DU–PW/PW/HTPB composites. The relevant results are presented in Fig. 6 and Table 1. As shown in Fig. 6a, there is one weight loss stage in the TGA curves of PW and DU–PW due to the evaporation and decomposition of PW and DU. Compared with PW, the *T*_1,0_, *T*_1,e_ and *T*_1,m_ of the DU–PW composite dramatically increased by 3.78, 75.63 and 51.31 °C, respectively, due to that the DU molecules could absorb and shape-stabilize PW to delay the evaporation of PW.^41^ The total weight loss of DU–PW was lower than that of PW due to the additional DU molecules. Thus, the total weight loss of DU–PW/HTPB was slightly lower than that of PW/HTPB. In the TGA curve of pristine HTPB, there are two decomposition stages, corresponding to the depolymerization and decomposition of HTPB.^42^ For the PW/HTPB composite sample, there are also two weight loss stages in the TGA curve. The first weight loss stage is attributed to the evaporation of PW and partial decomposition of HTPB. The second stage is ascribed to the decomposition of HTPB. Moreover, the introduction of PW in the PW/HTPB composite resulted in a decrease in the total weight loss compared with the HTPB matrix. It is noteworthy that the *T*_1,0_, *T*_1,e_ and *T*_1,m_ of the DU–PW/HTPB composite are higher than those of PW/HTPB as expected. Therefore, it was demonstrated that the introduction of DU–PW can improve the thermal stability of the PW/HTPB composite.

**Table 1:** Thermal stability of PW, DU–PW, pristine HTPB, PW/HTPB and DU–PW/HTPB composites^a^

Samples	Step 1				Step 2				Total weight loss (%)
	*T*_1,o_ (°C)	*T*_1,e_ (°C)	*T*_1,m_ (°C)	Weight loss (%)	*T*_2,o_ (°C)	*T*_2,e_ (°C)	*T*_2,m_ (°C)	Weight loss (%)	
PW	145.39	298.81	259.19	98.12	—	—	—	—	98.12
DU–PW	149.17	374.44	310.50	93.21	—	—	—	—	93.21
HTPB	212.83	402.83	324.83	28.02	418.83	511.25	461.17	68.33	96.35
PW/HTPB	208.33	316.83	395.17	43.14	403.17	490.44	460.83	52.43	95.57
DU–PW/HTPB	209.83	321.67	401.50	41.39	406.50	499.679	462.67	53.4	94.79

a *T*_1,0_ and *T*_1,e_ were determined from the cross-point of two tangent lines at related bending locations during the first rapid decomposition. *T*_1,m_ was determined as the temperature at the peak on the derivative curve of the first decomposition. Similar methods were adopted to obtain *T*_2,0_, *T*_2,e_ and *T*_2,m_.

### 3.6. Thermal reliability of DU–PW/PW/HTPB composites

**Fig. 7:**
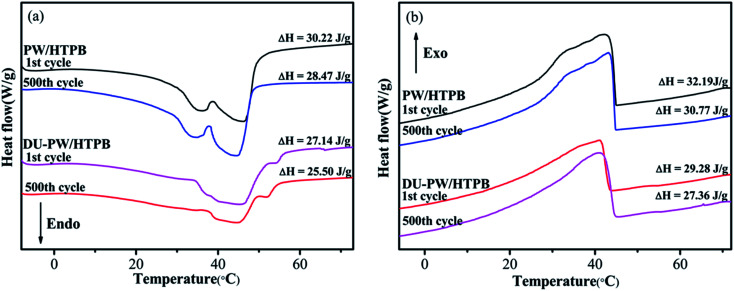
(a) Melting and (b) solidifying DSC curves of PW/HTPB and DU–PW/HTPB composites before and after 500-thermal-cycle test.

A 500-thermal-cycle test was carried out to investigate the thermal reliability of the DU–PW/HTPB composites. The DSC curves of PW/HTPB and DU–PW/HTPB composites before and after thermal cycling are shown in Fig. 7. As observed in Fig. 7, the temperatures at the melting and solidifying peaks of the PW/HTPB and DU–PW/HTPB samples are almost consistent before and after repetitive thermal cycles. The phase change enthalpies of the PW/HTPB and DU–PW/HTPB composites slightly decreased after 500-thermal cycles due to the diffusion of PW during the long-time thermal cycling process. This result illustrated that the DU–PW/HTPB composite exhibited good thermal reliability.

### 3.7. Leakage of DU–PW/PW/HTPB composites

**Fig. 8:**
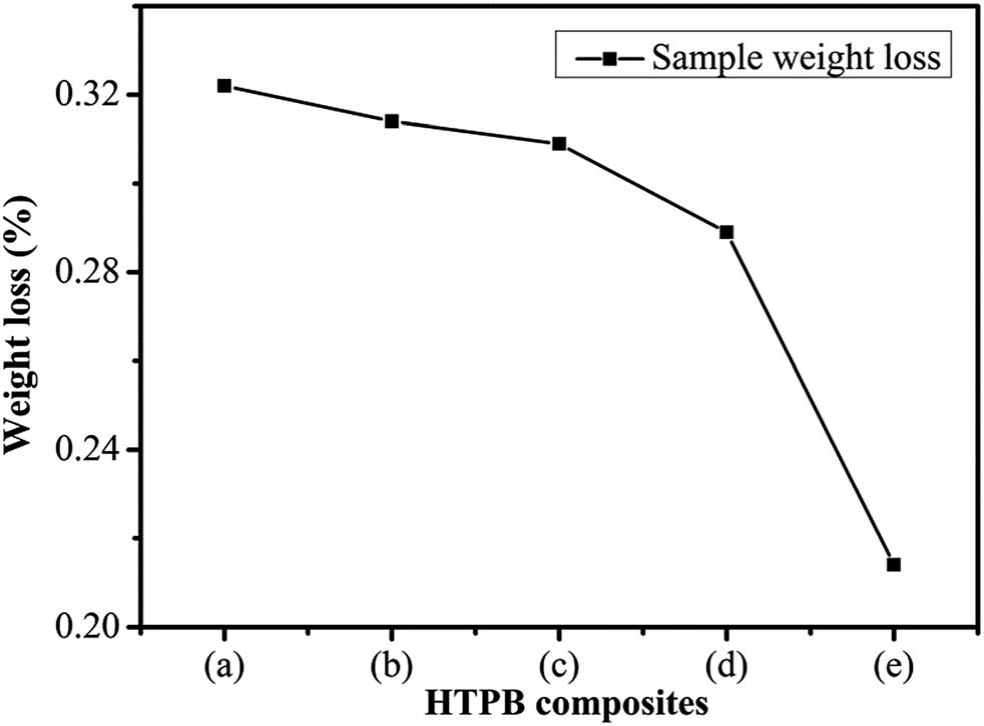
Sample weight loss of (a) PW/HTPB, (b) 20 wt% DU–PW/PW/HTPB, (c) 40 wt% DU–PW/PW/HTPB, (d) 60 wt% DU–PW/PW/HTPB, (e) DU–PW/HTPB composite.

The weight loss of the PW/HTPB, DU–PW/PW/HTPB and DU–PW/HTPB composites is shown in Fig. 8. As observed from the weight loss curve in Fig. 8, with increasing contents of DU–PW, the weight loss of DU–PW/PW/HTPB composites gradually decreased. The weight loss of samples reduced from 0.32 wt% for PW/HTPB to 0.21 wt% for DU–PW/HTPB composites. This indicated that the 3D structure of DU can shape-stabilize the PW firmly and prevent the PW molecules from moving out of the HTPB matrix as described in Fig. 1 and verified by Fig. 4. Thus, it was revealed that DU–PW can serve as a type of shape-stabilized PW in the HTPB matrix.

### 3.8. Mechanical properties of DU–PW/PW/HTPB composites

The results of compression and tensile tests for pristine HTPB, PW/HTPB, DU–PW/PW/HTPB and DU–PW/HTPB composites are presented in Table 2. As shown in Table 2, because PW always exhibited poor mechanical properties,^17^ the compressive modulus, compressive strain, tensile strength and elongation at break of PW/HTPB decreased compared with the HTPB matrix. With the increasing addition of DU–PW, the compressive modulus and compressive strength of DU–PW/PW/HTPB composites increased compared with PW/HTPB. The compressive modulus and compressive strength of DU–PW/HTPB were improved as much as 1.1× and 1.8× than those of PW/HTPB. Moreover, the tensile modulus, tensile strength and elongation at break of DU–PW/PW/HTPB composites were almost consistent, while higher than those of PW/HTPB. This indicated that DU–PW can provide mechanical reinforcement for the PW/HTPB composite, which may be attributed to the fact that the 3D structure of DU molecules can stabilize PW and firmly entangle with the HTPB matrix.

**Table 2:** Mechanical properties of pristine HTPB, PW/HTPB, DU–PW/PW/HTPB and DU–PW/HTPB composites

Samples	Compressive modulus^a^ (MPa)	Compressive strength^a^ (MPa)	Compressive strain^b^ (%)	Tensile modulus^c^ (MPa)	Tensile strength^d^ (MPa)	Elongation at break (%)
HTPB	0.0123 ± 0.0003	0.056 ± 0.004	87.30 ± 5.89	0.014 ± 0.003	0.264 ± 0.024	343.88 ± 26.047
PW/HTPB	0.0118 ± 0.0007	0.061 ± 0.004	84.62 ± 3.61	0.015 ± 0.005	0.252 ± 0.041	294.28 ± 35.79
20 wt% DU–PW/PW/HTPB	0.0132 ± 0.0006	0.150 ± 0.004	78.33 ± 0.87	0.018 ± 0.001	0.249 ± 0.021	378.19 ± 7.52
40 wt% DU–PW/PW/HTPB	0.0181 ± 0.003	0.155 ± 0.005	77.76 ± 0.34	0.019 ± 0.003	0.250 ± 0.034	407.94 ± 10.27
60 wt% DU–PW/PW/HTPB	0.0230 ± 0.0006	0.156 ± 0.007	78.43 ± 0.23	0.019 ± 0.002	0.230 ± 0.017	458.28 ± 18.58
DU–PW/HTPB	0.0243 ± 0.0007	0.172 ± 0.001	75.73 ± 0.58	0.018 ± 0.003	0.240 ± 0.017	464.65 ± 25.00

a At 25% strain.

b At limit stress.

c At 100% strain.

d At break.

## 4. Conclusions

In this study, a novel PW-based HTPB with DU–PW PCM was proposed for prevention of PW leakage. The chemical composition, morphology, phase change performance, leakage, thermal and mechanical properties of DU–PW and DU–PW/PW/HTPB composites were evaluated. From the results obtained, the main conclusion can be drawn is that the *T*_m_ and *T*_c_ of DU–PW were enhanced by 2.84 and 1.2 °C, respectively, compared with that of PW. DU–PW can be homogeneously dispersed in the HTPB matrix and effectively prevent the exudation of PW. With the addition of DU–PW, the composites can retain relatively high latent heat and good thermal reliability. Moreover, the thermal stability of DU–PW/HTPB was significantly improved compared with PW/HTPB. In addition, DU–PW can result in an enhancement of the compression properties of PW/HTPB. This indicated that the addition of DU–PW can improve the interaction between PW and the HTPB matrix. Consequently, DU–PW/PW/HTPB composites have a good prospect in application of PBX systems.

## Conflicts of interest

The authors declare that they have no conflict of interest.

## Supplementary Material

RA-008-C7RA10574F-s001
