# Supplementary material for: Thermal and mechanical reinforcement of a novel paraffin-based hydroxyl-terminated polybutadiene (HTPB) binder containing a three-dimension (3D) diurea–paraffin wax (DU–PW) for prevention of PW leakage
Source: RSC Adv. 2018 Jan 3;8(2):1047–54. doi: 10.1039/c7ra10574f (PMC9077001; doi:10.1039/c7ra10574f)
Supplement: RA-008-C7RA10574F-s001 [file RA-008-C7RA10574F-s001.pdf]

Electronic Supplementary Material (ESI) for RSC Advances.
This journal is © The Royal Society of Chemistry 2017

Supporting Information

Thermal and mechanical reinforcement of a novel paraffin-based HTPB binder containing 3D diurea-paraffin wax (DU-PW) PCM for prevention of PW leakage

Xia Gao[a,b], Tianbo Zhao[a,*], Guan Luo[b], Baohui Zheng[b], Hui Huang[b], Xue Han[a], Rui Ma[a], Yuqiao Chai[a]

[a]*Key Laboratory of Cluster Science of Ministry of Education, Beijing Institute of Technology, Beijing 100081, PR China*

[b]*Institute of Chemical Materials, China Academy of Engineering Physics (CAEP), Mianyang 621900, PR China*

[*]Corresponding author. Tel.: +86-10-81381389
E-mail address: bit_bipt@126.com

Notes and references

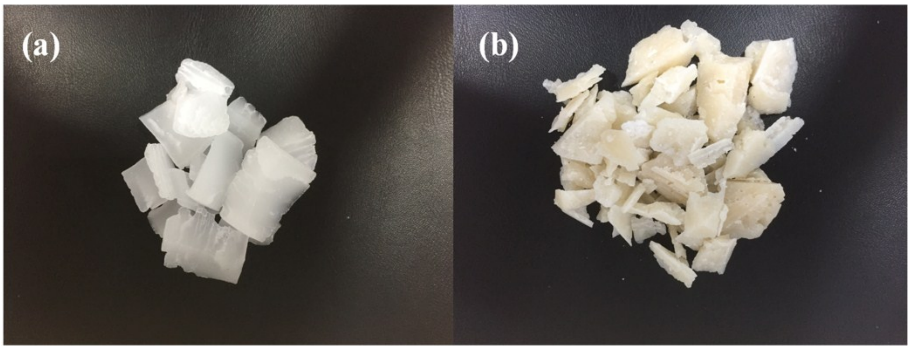

Fig. S1. Photographs of (a) pristine PW, (b) DU-PW composite.

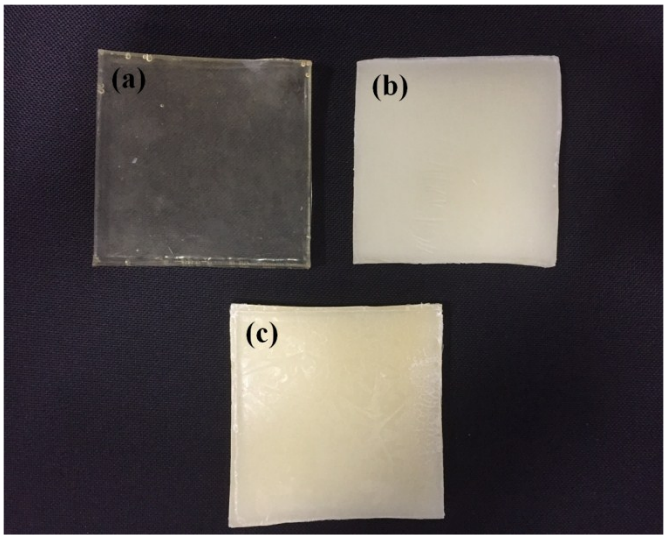

Fig. S2. Photographs of (a) pristine HTPB, (b) PW/HTPB composite, (c) DU-PW/HTPB composite.

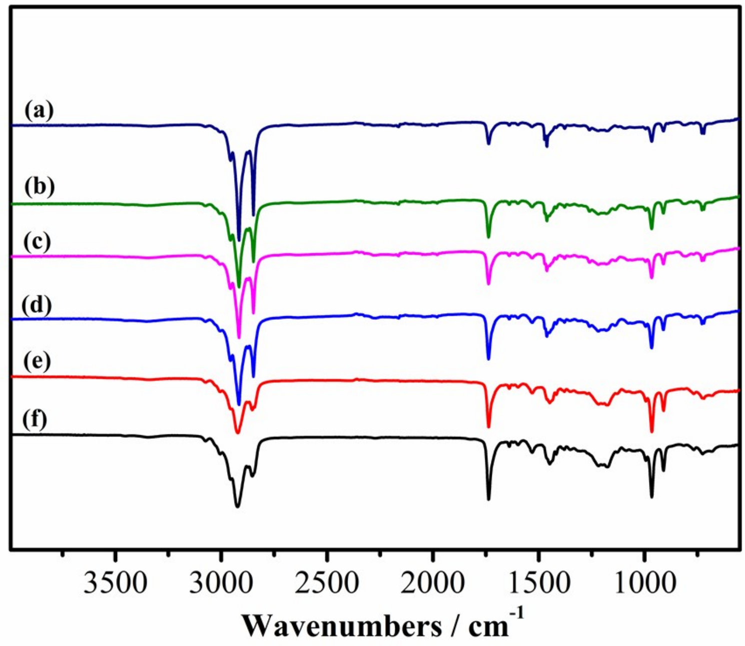

Fig. S3. FT-IR spectrum of (a) DU-PW/HTPB composite, (b) 60 wt% DU-PW/PW/HTPB, (c) 40wt% DU-PW/PW/HTPB, (d) 20 wt% DU-PW/PW/HTPB, (e) PW/HTPB composite and (f) pristine HTPB.

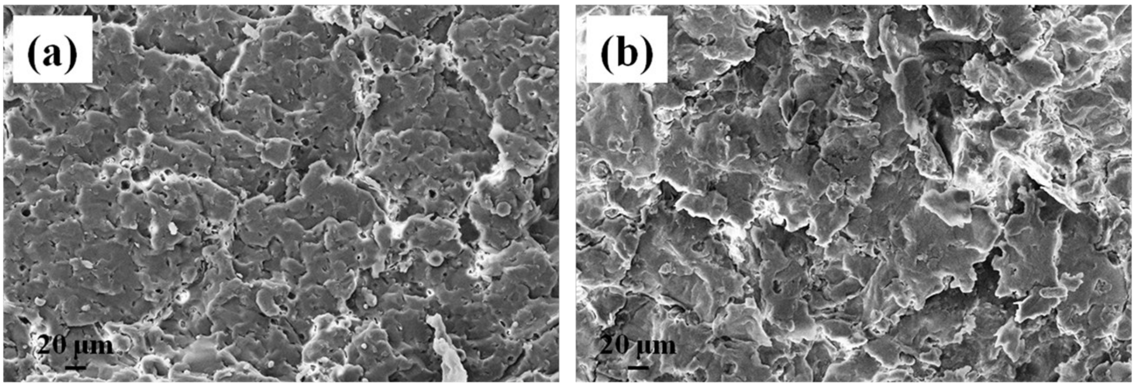

Fig. S4. SEM photographs of (a) 20 wt% DU-PW/PW/HTPB, (b) 40 wt% DU-PW/PW/HTPB composites.

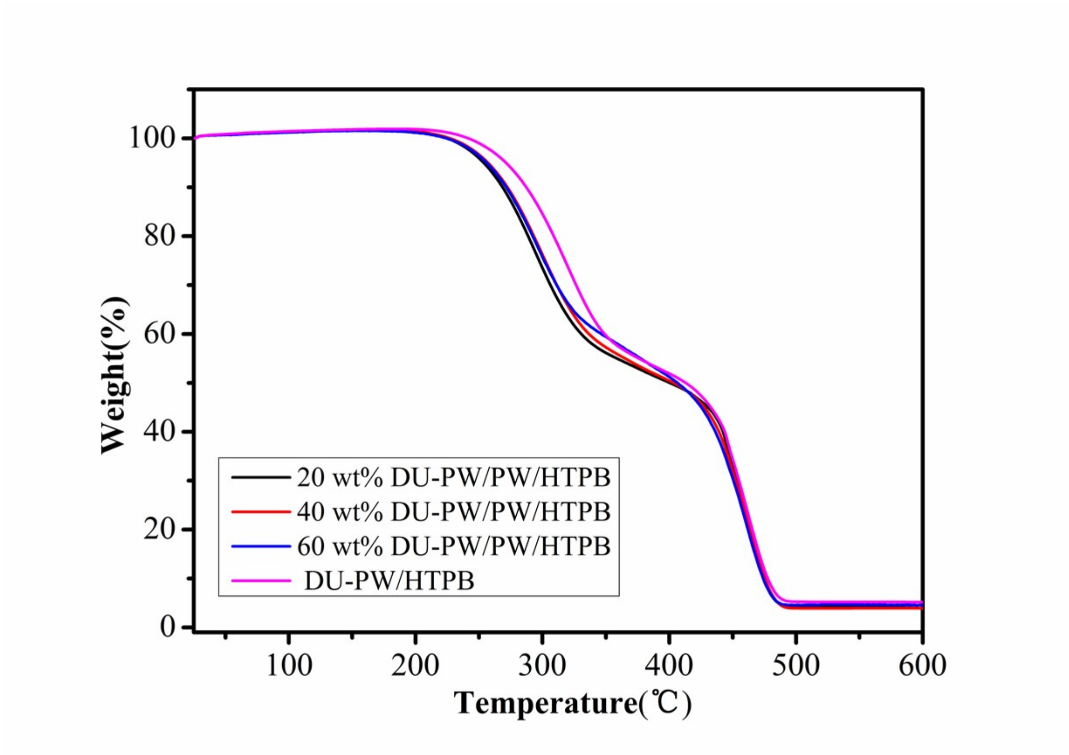

Fig. S5. TGA curves of 20 wt% DU-PW/PW/HTPB, 40 wt% DU-PW/PW/HTPB, 60 wt% DU-PW/PW/HTPB and DU-PW/HTPB composites.

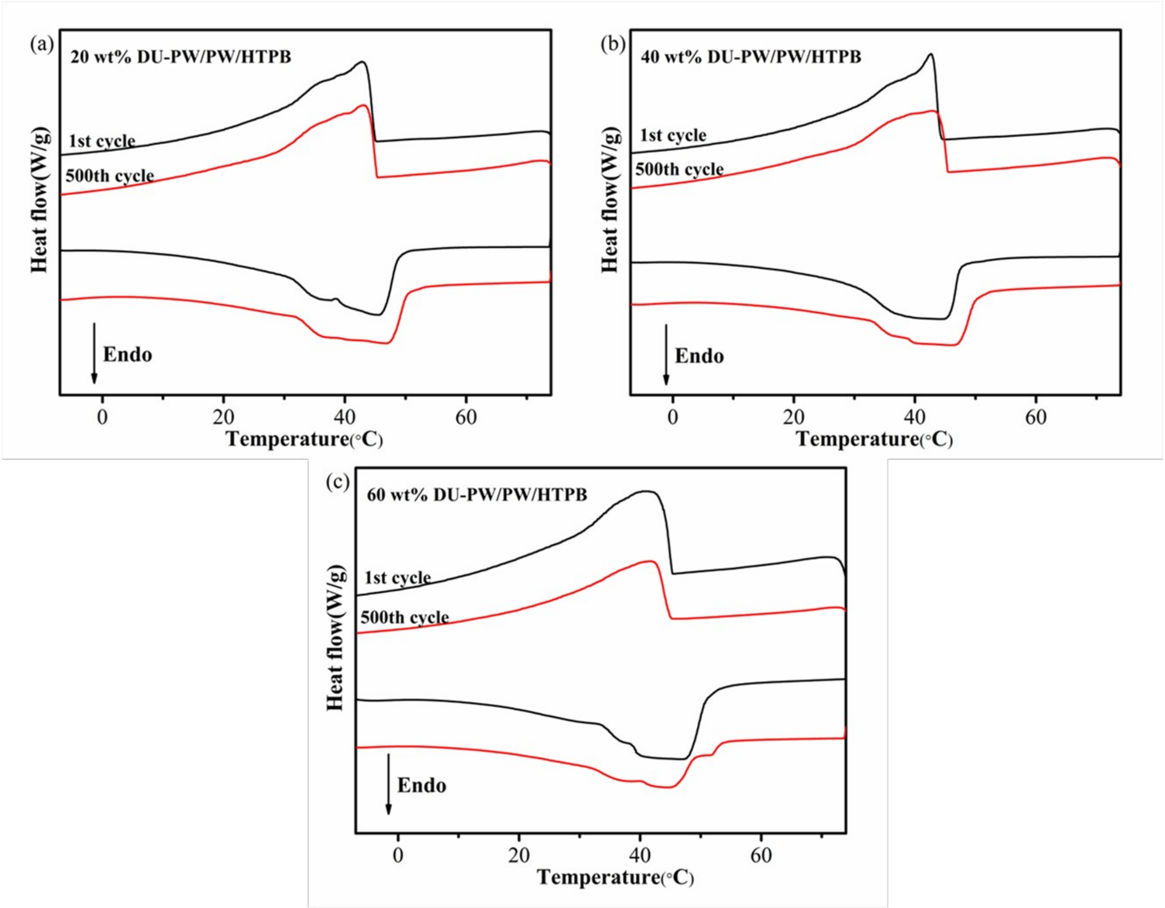

Fig. S6. DSC curves of (a) 20 wt% DU-PW/PW/HTPB, (b) 40 wt% DU-PW/PW/HTPB, (c) 60 wt% DU-PW/PW/HTPB composites before and after 500-thermal-cycle test.

Table S1. The recipe of DU-PW/PW/HTPB composites.

Samples	PW (g)	DU-PW (g)
HTPB	0	0
PW/HTPB	125	0
20 wt% DU-PW/PW/HTPB	100	25
40 wt% DU-PW/PW/HTPB	75	50
60 wt% DU-PW/PW/HTPB	50	75
DU-PW/HTPB	0	125

Table S2. DSC analysis data of PW, DU-PW, pristine HTPB and DU-PW/PW/HTPB composites with different contents of DU-PW.

Samples	Melting process		Freezing process		ΔT_s[a] (°C)	ΔH_1[b] (J/g)	ΔH_2[c] (J/g)	Error[d] (%)
	T_m (°C)	ΔH_m (J/g)	T_c (°C)	ΔH_c (J/g)				

HTPB	—	—	—	—	—	—	—	—
PW	49.61	167.80	48.35	168.10	1.26	167.95	—	—
DU-PW	52.45	143.23	49.55	144.30	2.90	143.77	—	—
PW/HTPB	46.29	30.22	42.08	32.19	4.21	31.21	33.59	7.09
20 wt% DU-PW/PW/HTPB	45.62	32.31	42.74	33.81	2.88	33.06	32.62	-1.35
40 wt% DU-PW/PW/HTPB	44.41	30.29	42.68	32.20	1.73	31.26	31.66	1.26
60 wt% DU-PW/PW/HTPB	43.40	28.52	42.74	30.47	0.66	29.50	30.69	3.88
DU-PW/HTPB	44.64	27.14	41.55	29.28	3.09	28.21	28.75	1.88

a: $\Delta T_s = T_c - T_m$. b: $\Delta H_1 = (\Delta H_c + \Delta H_m)/2$. c: Calculated enthalpy from the contents of the DU-PW/PW/HTPB composite. d: Error %=$(\Delta H_2 - \Delta H_1)/\Delta H_2$.
